# Development and validation of a social alienation predictive model for older maintenance hemodialysis patients based on latent profile analysis—a cross-sectional study

**DOI:** 10.1186/s12877-024-05116-9

**Published:** 2024-06-05

**Authors:** Guannan Wang, Jing Dong, Na Zhu, Yiping Zhu

**Affiliations:** 1 Hemodialysis Center, Li Huili Hospital, Ningbo Medical Center, No.57 Xingning Road, Ningbo, 315000 Zhejiang Province China; 2 Hemodialysis Center; Medical and Health Group, First People’s Hospital of Xiangshan County, No.291 Dandong Street, Xiangshan County, Ningbo, 315700 Zhejiang Province China

**Keywords:** Maintenance hemodialysis, Social alienation, Latent profile analysis, Predictive model

## Abstract

**Background:**

Social alienation refers to the state of feeling isolated, helpless, and unsatisfied due to maintaining distance from others or avoiding social interaction and activities. This phenomenon is caused by a lack of social skills, social anxiety, physical health problems, and other reasons. Older maintenance hemodialysis patients are exposed to a higher risk of social alienation. However, previous studies have been performed using the total score of the scale, which does not allow the identification of the characteristics of various patient groups with different levels of social alienation. In contrast, latent profile analysis can classify individuals into different categories based on continuous observational indicators, which improves accuracy and provides a more objective assessment by accounting for the uncertainty of variables. Given the concealed nature of social alienation and the differences in characteristics and treatment measures between different profiles, developing a predictive model for social alienation in older maintenance hemodialysis patients holds significance.

**Objective:**

To explore the latent profile analysis of social alienation in older maintenance hemodialysis patients and to develop and validate a predictive model for social alienation in this population.

**Methods:**

A total of 350 older maintenance hemodialysis patients were selected as the study subjects using convenience sampling. A cross-sectional survey was conducted using a general information questionnaire, the Generalized Alienation Scale, and the Self-Perceived Burden Scale. Based on the results of the Generalized Alienation Scale, a latent profile analysis was performed, followed by univariate analysis and multinomial logistic regression to develop a predictive model. The effectiveness of the predictive model was evaluated in terms of its authenticity, reliability, and predictive ability.

**Results:**

Three hundred nineteen valid questionnaires were collected. The social alienation of older maintenance hemodialysis patients based on latent profile analysis were divided into three profiles, which were named the low/medium/high-symptom groups, comprising 21%, 38.9%, and 40.1% of participants, respectively. Based on male, monthly social activity hours, Age-Adjusted Charlson Comorbidity Index, dialysis age, and Self-Perceived Burden Scale, a predictive model of social alienation for older maintenance hemodialysis patients was developed, and the Hosmer–Lemeshow tests showed no statistical significance (*P* > 0.05). The model has high predictive efficiency in authenticity, reliability and predictability.

**Conclusion:**

Older maintenance hemodialysis patients exhibited moderate to high levels of social alienation. The latent profile analysis based method was used to divide patients into low/medium/high-symptom profiles, and the predictive model demonstrates excellent authenticity, reliability, and predictability.

## Background

Social alienation refers to the state of isolation, helplessness, and dissatisfaction experienced by individuals due to social interaction avoidance, which may be attributed to a lack of social skills, social anxiety, physical health problems, and other reasons [1]. Social alienation is detrimental to the health of the older adults, leading to physical function decline, dementia, type II diabetes, loneliness, depression, and other psychological problems, and is an independent risk factor for death [2, 3]. With the aging population in China and the accessibility to blood dialysis technology, the number of maintenance hemodialysis (MHD) patients over the age of 60 has grown significantly, accounting for nearly 70% [4]. However, older MHD patients have different psychological status and physical functions than young and middle-aged MHD patients. An even greater difference was observed in patients with poorer physical function, longer dialysis age, underlying diseases, and complications.

Numerous studies have examined the social alienation of the older adults, Wong et al.investigated the social alienation of 37 Hong Kong older people and divided them into three categories: unease about aging, helplessness as the older adults and anger at the current situation [5]. Liu et al. conducted a study on social alienation in 322 older patients with coronary heart disease, utilizing latent profile analysis (LPA) to categorize them into low social alienation groups (31.9%), moderate social alienation-meaninglessness group (26.5%) and high social alienation group (41.6%), revealing differences in coping styles among different categories of patients [6]. However, few studies have been conducted on social alienation in MHD patients, and existing studies have focused on risk factors, adverse outcomes, and intervention approaches in this population [7, 8]. The social alienation of MHD patients, especially the older patients, involves psychological, physiological, family, social and economic factors [7]. Even if the degree of disease progression is the same among individuals, the differences in their family, community and individual cognition and self-regulation levels of the disease will lead to psychological distress and social function degradation of patients to varying degrees, Just as Zhu et al. revealed differences in Generalized Alienation Scale scores among different characteristic populations [8]. For example, some individuals still feel lonely although they have rich social networks, some individuals are often isolated although they have strong social willingness, while some patients are self-isolated due to frustration after illness. Although these factors will lead to social alienation, the underlying causes and coping strategies vary. Therefore, it is necessary to further quantify the scores of each dimension of social alienation of MHD patients, and comprehensively analyze the characteristics of each dimension of patients to finely divide different categories of patients, laying a foundation for follow-up individualized treatment and nursing.

In response to these limitations, Latent class model has been increasingly used in different areas of research, including education, sociology, and psychology[9]. Latent class model is a method that distinguishes individuals' potential characteristic classifications based on their responses to external measurement items. The characteristics of different groups of subjects were divided into latent class analysis suitable for classified variables and LPA suitable for continuous variables according to the type of external variables [10]. LPA is an analysis method that uses probability estimation and comparison for classification under a probability model, determining each class through fitting index and statistical test [11]. This method takes individuals as the center and explains the relationship between external continuous variables through latent class variables, and divides them into different classes according to their characteristics, thereby identifying independent classes [12]. It not only considers the uncertainty of variables but also classifies them more objectively and results more accurately.

Given the complexity and differences in characteristics and treatment measures between various levels of social alienation, identifying and effectively minimizing risk factors at an early stage holds significance. Therefore, this study aimed to explore the latent profile analysis of older MHD patients, develop and validate a predictive model for social alienation in this population, and provide a reference for subsequent targeted intervention programs.

## Methods

### Study design and participants

**Fig. 1:**
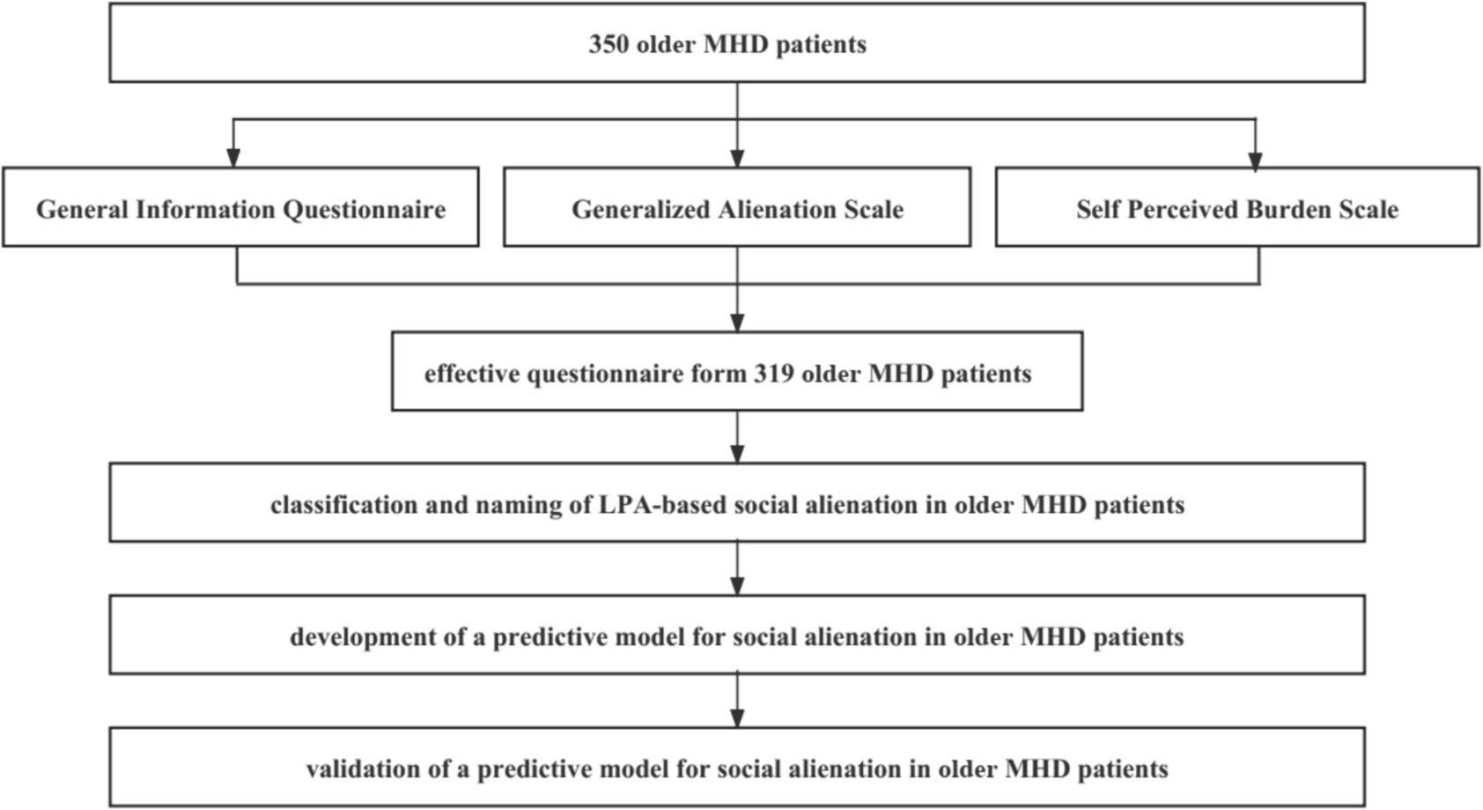
Flow diagram

This study was approved by the ethics committee of Lihuili Hospital of Ningbo Medical Center (No.: 2023224). 350 MHD patients admitted to the hemodialysis center of the hospital from August 2023 to December 2023 were selected by convenience sampling as the study subjects. The inclusion criteria were: age ≥ 65 years; diagnosed with end-stage renal disease and receiving regular hemodialysis treatment for ≥ 3 months; clear consciousness. The exclusion criteria were: suffering from mental illness; cognitive dysfunction; severe disease exacerbation; hearing or visual impairment; transferring to other hospitals or receiving kidney transplantation during the study period. According to Kendall's method for sample size estimation, a total of 28 variables were collected in this study (including 21 general information and disease-related variables, 4 dimensions in the Generalized Alienation Scale, and 3 dimensions in the Self-Perceived Burden Scale) [13]. The required sample size was calculated as 10 ~ 20 times the variable number. Considering a 10% ineffective rate, at least 312 samples should be included. In this survey, questionnaires were distributed to 350 MHD patients and questionnaires were effectively recovered from 319 MHD patients, with a recovery rate of 91.1% (Fig. 1).

### Measures

#### General information questionnaire

The general information questionnaire was designed by the research team and included demographic data such as gender, age, body mass index (BMI), residence (rural/urban), education years, family members living together, monthly social activity hours (participating in volunteer activities, public welfare activities, square dancing, ball games, and other social activities), monthly income, monthly out-of-pocket medical expenses (excluding medical insurance payments), and other comorbidities. Disease-related data such as Age-Adjusted Charlson Comorbidity Index (ACCI), dialysis age, type of vascular access (autogenous arteriovenous fistula, central venous catheters and arteriovenous graft), biochemical indicators (calcium, phosphorus, hemoglobin, albumin, B-type natriuretic peptide), and primary disease (chronic glomerulonephritis, hypertension nephropathy, diabetic nephropathy) were also collected.

#### Generalized Alienation Scale (GAS)

Wu et al. conducted a cross-cultural adaptation based on the GAS developed by Jessor to better assess the social alienation among the Chinese older population, with a Cronbach's α coefficient of 0.77 [14, 15]. The scale consists of 15 items covering four dimensions: other' alienation, suspicion, self-alienation, and meaninglessness. Each item uses a one-point (very disagree) to four-point (very agree) Likert scale for scoring. The total score ranged from 15 ~ 60 points, with higher scores indicating stronger social alienation tendencies. In this study, the Cronbach's α coefficient of this scale was 0.801, each dimension ranged from 0.73 for suspicion to 0.86 for self-alienation.

#### Self-Perceived Burden Scale (SPBS)

This scale was compiled by Cousineau and translated into Chinese by Zhang, to evaluate the patient's perceived burden level [16, 17]. The scale consists of 10 items, including three dimensions: physical burden (5 items), emotional burden (4 items), and economic burden (1 item). Using a Likert 5-point scoring method, the questions were scored from 1 ~ 5 points to indicate never, occasionally, sometimes, frequently, and always, respectively. The scale has a total score of 10 ~ 50 points. SPBS < 20 points suggest no significant self perceived burden, 20 ~ 29 points indicate mild self perceived burden, 30 ~ 39 points indicate moderate self perceived burden, and ≥ 40 points suggest severe self perceived burden. The Cronbach's α coefficient of the Chinese version of the scale was 0.85. In this study, Cronbach's α coefficient of this scale was 0.83, each dimension ranged from 0.74 for physical burden to 0.88 for economic burden.

### Quality control

The study subjects were screened according to inclusion and exclusion criteria. During the dialysis treatment, the researchers used unified instruction language and collected data in a face-to-face manner. The questionnaire was filled out for 10 ~ 20 min and then checked to avoid omissions. Any queries regarding the filled questionnaires were verified with the survey subjects immediately. All data were entered into Excel in parallel double-entry mode and the logic was checked for correctness.

### Statistical analysis

Mplus8.0 software was used for LPA, and 1 ~ 5 latent profiles were fitted respectively. Model fit indices included Akaike Information Criterion (AIC), Bayesian Information Criterion (BIC), adjusted Bayesian (aBIC), Entropy, Lo-Mendell-Rubin adjusted likelihood Ratio Test (LMRT), and Bootstrap Likelihood Ratio Test (BLRT). Lower values of AIC, BIC, and aBIC indicate better model fit. Entropy is an indicator of classification accuracy and has values ranging from 0 ~ 1, with values closer to 1 indicating more accurate classification. LMRT and BLRT were used to compare the fit differences between the K-1 category and K category models, with significant differences indicating that the K-profile model is superior to the K-1 profile model, where K represents the number of freely estimated parameters.

SPSS 27.0 software was used for statistical analysis. Normally distributed data with homogeneous variance were represented as mean ± standard deviation; otherwise, the median (IQR) was used. Frequency (%) was used for count data. The R*C table chi-square test or Fisher exact probability method was used to compare multiple grouping variables among different categories. In addition, the Bonferroni adjustment *P* value was used for pairwise comparison, with *P* ≤ 0.017 considered statistically significant. Multinomial logistic regression analysis was used to identify risk factors for latent profile of social alienation in older MHD patients and construct a predictive model. Subsequently, the Hosmer–Lemeshow test was performed to evaluate the goodness-of-fit of the predictive model. In this study, *P* < 0.05 was considered statistically significant except for pairwise comparisons.

## Results

### Classification and naming of LPA-based social alienation in older MHD patients

Taking the four dimensions of GAS as explicit variables, gradually fitting the number of latent profiles from 1 to 5. When three profiles were retained, the values of AIC, BIC, and aBIC were lower, and the value of Entropy was higher, indicating that the model had high classification accuracy. Moreover, both LMR and BLRT were significant (*P* < 0.001). Therefore, three profiles were retained, as shown in Table 1. Furthermore, the probability of correct classification for each category was calculated as 94.2%, 88.1%, and 95.1%, respectively. The results verified the reliability of the classification and latent profile analysis, as shown in Table 2. Patients in C1 scored low on all four dimensions and were named the low-symptom group, accounting for 40.1% (128 cases); patients in C2 scored moderately between C1 and C3 on all four dimensions and were named the medium-symptom group, accounting for 38.9% (124 cases); patients in C3 scored high on all four dimensions and were named the high-symptom group, accounting for 21% (67 cases).

**Table 1:** Fit indices for five models using latent profile analysis (*N* = 319)

Profile	AIC	BIC	aBIC	*p*LMR	*p*BLRT	Entropy	Class Probability
1-profile	8648	8751	8614	-	-	-	1
2-profile	8015	8122	7933	< 0.001	< 0.001	0.931	0.166/0.834
3-profile	7351	7416	7387	< 0.001	< 0.001	0.933	0.210/0.389/0.401
4-profile	7133	7238	7178	0.072	< 0.001	0.920	0.159/0.332/0.313/0.079
5-profile	7011	7191	7019	0.059	< 0.001	0.911	0.255/0.112/0.274/0.321/0.038

**Table 2:** Probability of correct classification for each category (*N* = 319)

Profile	C1	C2	C3
C1	0.942	0.033	0.025
C2	0.067	0.881	0.052
C3	0.027	0.021	0.951

C1, low-symptom group; C2, medium-symptom group; C3, high-symptom group

### Comparison of baseline data among the three groups of older MHD patients after LPA

Thirteen variables, including male, age, education years, and family members living together, showed statistically significant differences among the three groups of older MHD patients (*P* < 0.05), while other variables showed no statistical differences (*P* > 0.05), as shown in Table 3.

**Table 3:** Comparison of baseline data among the three profiles of older MHD patients (*N* = 319)

Item	C1 (*n* = 128)	C2 (*n* = 124)	C3 (*n* = 67)	^2^/F	P
male(%)	60 (46.9)	70 (56.4) ^a^	44 (65.7) ^a^	6.243	0.044
age	71.1 ± 6.5	71.4 ± 6.3	74.9 ± 6.7^ab^	8.567	< 0.001
body mass index	24.2 ± 4.2	24.3 ± 4.6	24.7 ± 4.8	0.346	0.708
residence [urba(%)]	86 (67.2)	78 (63.0)	43 (64.2)	0.526	0.769
education years	7.9 ± 3.8	6.5 ± 3.6^a^	5.1 ± 3.5^ab^	14.210	< 0.001
family members living together	1.6 ± 0.8	1.3 ± 0.9^a^	0.8 ± 1.0^ab^	20.386	< 0.001
monthly social activity hours	11.8 ± 8.2	9.5 ± 5.6^a^	5.0 ± 3.3^ab^	23.942	< 0.001
monthly income	6031.4 ± 301.3	5521.3 ± 356.1^a^	4251.1 ± 161.6^a^	10.680	< 0.001
monthly out-of-pocket medical expenses	696.8 ± 140.0	749.2 ± 185.7	925.3 ± 328.1^ab^	25.536	< 0.001
combined cardiovascular system diseases[case(%)]	71 (55.5)	69 (55.6)	37 (55.2)	0.003	0.998
combined endocrine system diseases[case(%)]	25 (19.5)	21 (17.0)	17 (25.4)	1.960	0.375
combined respiratory system diseases[case(%)]	35 (27.3)	36 (29.0)	24 (35.8)	1.566	0.457
Age-Adjusted Charlson Comorbidity Index	6.5 ± 1.1	6.5 ± 1.0	7.0 ± 1.4^ab^	5.883	0.003
dialysis age	5.9 ± 0.8	6.4 ± 1.2^a^	6.9 ± 1.3^ab^	17.029	< 0.001
vascular access[case(%)]				0.669	0.978
autogenous arteriovenous fistula	102 (79.7)	95 (76.6)	54 (80.6)		
central venous catheters	23 (18)	26 (21)	12 (17.9)		
arteriovenous graft	3 (2.3)	3 (2.4)	1 (1.5)		
calcium (mmol/L)	2.7 ± 0.4	2.5 ± 0.4	2.6 ± 0.4	2.103	0.124
phosphorus (mmol/L)	1.5 ± 0.3	1.5 ± 0.3	1.4 ± 0.3	0.245	0.783
hemoglobin (g/L)	112.1 ± 12.6	112.7 ± 12.5	110.9 ± 12.1	0.470	0.625
albumin (g/L)	39.4 ± 5.1	39.8 ± 5.3	38.7 ± 4.8	0.936	0.393
B-type natriuretic peptide (pg/mL)	55.3 ± 21.0	56.7 ± 21.0	51.8 ± 20.0	1.234	0.293
primary disease				5.510	0.239
chronic glomerulonephritis[case(%)]	71 (55.5)	78 (62.9)	45 (67.2)		
hypertension nephropathy[case(%)]	25 (19.5)	27 (21.8)	16 (23.9)		
diabetic nephropathy[case(%)]	28 (21.9)	23 (18.5)	6 (9.0)		
Self-Perceived Burden Scale	20.3 ± 2.9	33.7 ± 1.6^a^	42.3 ± 1.7^ab^	2445.4	< 0.001
physical burden	10.8 ± 2.5	16.4 ± 1.1^a^	23.4 ± 1.8^ab^	1000.2	< 0.001
emotional burden	7.5 ± 1.1	14.6 ± 1.1^a^	16.4 ± 1.7^ab^	1470.6	< 0.001
economic burden	1.9 ± 1.0	2.7 ± 1.1^a^	2.5 ± 1.3^a^	15.086	< 0.001

a statistically significant difference from C1 (*P* < 0.05), b statistically significant difference from C2 (*P* < 0.05), C1 low-symptom group, C2 medium-symptom group, C3 high-symptom group

### Generalized alienation scale of older MHD patients

The social alienation of the older MHD population was at a moderate to high level. The scores of others' alienation, self-alienation, and meaninglessness in the high-symptom group were higher than those in the other two groups (*P* < 0.05), and the score of suspicion was higher than that in the low-symptom group (*P* < 0.05). However, no difference was found between the medium-symptom group and the low-symptom group (*P* > 0.05), as shown in Table 4.

**Table 4:** Generalized alienation scale of older MHD patients

Dimension	Item	Dimension score	Item score	C1	C2	C3	F	P
Generalized Alienation Scale	15	40.8 ± 6.0	2.7 ± 0.5	35.3 ± 2.8	42.0 ± 2.5^a^	49.2 ± 4.0^ab^	490.461	< 0.001
other' alienation	5	12.9 ± 2.4	2.7 ± 0.3	11.3 ± 1.6	12.8 ± 1.5^a^	15.9 ± 2.0^ab^	164.357	< 0.001
suspicion	4	11.1 ± 2.7	2.6 ± 0.3	8.8 ± 2.1	12.4 ± 1.7^a^	12.9 ± 1.8^a^	151.259	< 0.001
self-alienation	3	8.1 ± 1.5	2.7 ± 0.5	7.0 ± 0.8	8.3 ± 1.1^a^	9.8 ± 1.4^ab^	147.063	< 0.001
meaninglessness	3	8.7 ± 1.5	2.8 ± 0.4	8.1 ± 0.8	8.4 ± 1.1^a^	10.4 ± 1.9^ab^	86.613	< 0.001

Note: a, statistically significant difference from C1 (*P* < 0.05); b, statistically significant difference from C2 (*P* < 0.05); C1, low-symptom group; C2, medium-symptom group; C3, high-symptom group

### Multinomial logistic regression

Taking C1 as control group, variables with statistical differences were further subjected to multinomial logistic regression analysis. Finally, male (X_1_), monthly social activity hours (X_2_), ACCI (X_3_), dialysis age (X_4_), and SPBS (X_5_), were independent risk factors for social alienation of older MHD patients (*P* < 0.05), see Table 5.

**Table 5:** Multinomial logistic regression

	Item	B	SE	Wald	P	*OR* (95% CI)
C3	intercept	-50.482	12.720	25.110	< 0.001	-
	male	0.120	0.030	4.623	< 0.001	1.134 (1.07 ~ 1.19)
	monthly social activity hours	-0.173	0.086	4.107	0.041	0.841 (0.711 ~ 0.994)
	ACCI	0.486	0.094	26.803	< 0.001	1.626 (1.352 ~ 1.954)
	dialysis age	0.100	0.020	4.630	< 0.001	1.110 (1.06 ~ 1.15)
	SPBS	1.100	0.300	3.700	< 0.001	1.201 (1.08 ~ 1.37)
C2	intercept	-8.081	2.036	4.102	< 0.001	-
	male	0.300	0.031	8.503	< 0.001	1.35 1(1.26 ~ 1.44)
	monthly social activity hours	-0.101	0.042	-2.241	0.025	0.912 (0.83 ~ 0.99)
	ACCI	0.514	0.073	6.972	< 0.001	1.661 (1.44 ~ 1.91)
	dialysis age	0.203	0.052	3.59	< 0.001	1.223 (1.09 ~ 1.35)
	SPBS	0.148	0.044	4.551	< 0.001	1.115(1.08 ~ 1.21)

C2 medium-symptom group, C3 high-symptom group

### Development of the model

The results of the multinomial logistic regression show that the model was as follows:C3 = -50.482 + 0.12X_1_-0.173X_2_ + 0.486X_3_ + 0.1X_4_ + 1.1X_5_; C2 = -8.081 + 0.3X_1_-0.101X_2_ + 0.514X_3_ + 0.203X_4_ + 0.148X_5_, C1 = 0 (control group), Hosmer–Lemeshow test showed no statistical significance (χ^2^ = 10.781, *P* = 0.952), indicating a good model fit.

### Validation of the model

In the multinomial logistic regression analysis, selecting Estimated response probabilities, Predict categories, Predict category probability and Actual category probability options can bring the above model into the original dataset for internal verification. Comparing the predicted results with the actual results, the results suggested that the model has higher predictive efficiency for C1, C2 and C3 in authenticity (sensitivity, specificity, Youden index), reliability (accuracy, F1 score) and predictability (positive predictive value, negative predictive value),Table 6.

**Table 6:** Effectiveness Evaluation of the Model

Item	C1	C2	C3
Sensitivity	0.805	0.847	0.881
Specificity	0.791	0.831	0.905
Accuracy	0.796	0.837	0.899
Youden index	0.596	0.678	0.786
F1 score	0.800	0.842	0.889
positive predictive value	0.720	0.761	0.711
negative predictive value	0.858	0.895	0.966

C1 low-symptom group, C2 medium-symptom group, C3 high-symptom group

## Discussion

### The heterogeneity of latent profile characteristics of social alienation in older MHD patients

This study revealed moderate to high social alienation in older MHD patients compared to other populations. The patients were divided into three groups after LPA, namely the low/medium/high-symptom groups. The heterogeneity across groups was confirmed, which provides a basis for implementing individualized interventions for different categories.

Patients in the high-symptom group experienced higher feelings of alienation from others, self-alienation, and senselessness compared to the other two groups. They exhibited higher suspicion than the low-symptom group but showed no significant difference compared to the medium-symptom group. These results indicated that patients in this group face intense painful emotions and have more social and health risk factors, leading to a decline in their life and social skills. They require high attention. The medium-symptom group demonstrated scores between the high-symptom group and the low-symptom group across the four dimensions of social alienation. Social alienation in older MHD patients may be attributed to frequent travels between their homes and hospitals, limiting the time for social interaction between individuals and others, resulting in a sense of distance and loneliness [18]. Moreover, existing or potential health problems in older MHD patients often require others' care, including family members and social environments, leading to a lack of goals and meaning in life [19, 20]. Coupled with the expensive cost of hemodialysis, some patients require financial assistance. These factors directly lead to a sense of inferiority, negative self-evaluation, uncertainty about self-identity, and questioning of social values by individuals. Therefore, support networks including healthcare professionals, family members, friends, community organizations, and volunteers should be established to support patients in the medium/high-symptom groups, providing psychological support and promoting social participation. In addition, it is necessary to cultivate their interests and hobbies, encourage communication with others, and provide professional psychological intervention.

### Predictive model for social alienation in older MHD patients

The potential risk factors for predictive of social alienation in older MHD patients include male, low monthly social activity hours, high ACCI, long dialysis age, and high SPBS. Grothe reported that women harbored a more extensive and diverse social network than men, and were more willing to communicate with others when facing stress [21]. Communicating with others can help reduce the risk of social alienation, especially with the rise of group exercise activities such as square dance in recent years in Ningbo. However, in traditional Chinese culture, men exhibit independent and strong temperaments and are responsible for the economy. Older male MHD patients tend to face their diseases and expensive hemodialysis alone, which leads to self-doubt and denial, increasing the risk of social alienation [22].

Low monthly social activity hours are a risk factor for social alienation in older MHD patients. The longer the time spent participating in social activities, the more frequent interaction with others, leading to a sense of self-worth and reducing negative emotions. Such activities help patients adapt to the social changes caused by maintenance hemodialysis, reducing the risk of social alienation [23, 24]. The low monthly social activity hours in older MHD patients may be related to insufficient social participation conditions, limited social activity opportunities and social networks. Therefore, community organizations should host activities such as chess games to improve the overall level of community health services. In addition, dialysis centers can also regularly hold various forms of exchanges to share information and provide psychological and social support. Moreover, the relevant government departments can also promote older adults entertainment and sports activities to increase social interactions among older people and reduce the risk of social alienation.

ACCI is a quantitative scoring system for complications based on the number and severity of diseases experienced by patients [25]. MHD patients have a high incidence of complications (59.4%), often accompanied by systemic inflammation, malnutrition, and poor prognosis. Patients experience significant physical and psychological burdens, increasing the risk of social alienation compared to other populations [26, 27]. Therefore, careful assessment of symptom burden is needed for patients with high ACCI scores. Clinicians should pay attention to the types, severity, prognosis, and symptoms of multiple diseases when evaluating the risk of social alienation in patients with high ACCI scores.

As dialysis age increases, the physiological function of older MHD patients gradually declines, leading to frailty and sarcopenia [28, 29]. Subsequently, patients actively or passively reduce their social network, increasing the risk of social alienation. Patients with high SPBS are worried about imposing a burden on others due to their illness. As a result, these patients are reluctant to communicate about their illness with others and lack the tools to alleviate their concerns about their illness through various channels. They are easily immersed in negative emotions and adopt negative coping strategies [30]. Therefore, healthcare providers should regularly assess the level of self-perceived burden in older MHD patients and provide personalized guidance.

## Limitations

This study used a convenience sampling method and only surveyed patients from a single center, which may limit the representativeness of the sample. The questionnaire method can only provide superficial insights into the social alienation status of older MHD patients, and future studies should combine qualitative research to gain a deeper understanding of their social alienation. This study exclusively relies on empirical sample size calculations due to the limitations of the PASS software in determining sample size for multivariate regression analysis [13]. The applicability of the predictive model may be limited as it has only been internally validated and not externally validated prospectively. Furthermore, the diverse causes of social alienation and potential associations between various risk factors were not fully explored using traditional statistical methods, which poses limitations in screening risk factors and providing individualized disease predictives. Future studies should apply machine learning techniques to fine-tune and validate the model from multiple dimensions.

## Conclusion

Older maintenance hemodialysis patients exhibited moderate to high levels of social alienation. The latent profile analysis based method was used to divide patients into low/medium/high-symptom profiles, and the predictive model demonstrates excellent authenticity, reliability, and predictability.

## Data Availability

All data generated or analysed during this study are included in this article and its supplementary information files.

## Abbreviations

MHD: Maintenance hemodialysis
LPA: Latent profile analysis
BMI: Body mass index
ACCI: Age-adjusted charlson comorbidity index
GAS: Generalized alienation scale
SPBS: Self-perceived burden scale
